# Diagnostic performance of the gallbladder reporting and data system combined with color doppler flow imaging for gallbladder cancer in the Asian population

**DOI:** 10.3389/fonc.2024.1367351

**Published:** 2024-04-15

**Authors:** Rongling Wang, Lin Lv, Li Li

**Affiliations:** Department of Ultrasound, Qilu Hospital of Shandong University (Qingdao), Qingdao, Shandong, China

**Keywords:** GB-RADS, gallbladder cancer, ultrasound, CDFI, diagnostic performance

## Abstract

**Purpose:**

Evaluating the performance of the Gallbladder Reporting and Data System (GB-RADS) combined with Color Doppler Flow Imaging (CDFI) for the diagnosis of gallbladder wall thickening disease in an Asian population.

**Methods:**

In this study, the lesions were classified and the actual incidence rate of malignant tumors was calculated for each GB-RADS category, following the guidelines provided by GB-RADS. To evaluate the diagnostic performance of GB-RADS and GB-RADS combined with CDFI, we plotted Receiver Operator Characteristic (ROC) curves. The sensitivity (SE), specificity (SP), positive predictive value (PPV), negative predictive value (NPV), and accuracy (AC) were also calculated. Inter-observer agreement (IRA) between the two observers was assessed using Kappa values.

**Results:**

The incidence of malignancy risk for GB-RADS 2, 3, 4, and 5 was 9%, 12.5%, 72.2%, and 100%. The AUC for GB-RADS was 0.855 (95% CI: 0.800-0.900), with a sensitivity of 82.5%, a specificity of 84.6%, and an accuracy of 83.8%. The AUC of GB-RADS combined with CDFI was 0.965 (95% CI: 0.930-0.985), with a sensitivity of 96.2%, a specificity of 94.6%, and an accuracy of 95.2%. The AUC, sensitivity, specificity, and accuracy of GB-RADS combined with CDFI for diagnosing gallbladder malignancy were higher than those of GB-RADS alone, and the differences were statistically significant (all P < 0.05). The IRA was excellent between the two observers (Kappa = 0.870).

**Conclusions:**

GB-RADS combined with CDFI demonstrated excellent diagnostic accuracy when it comes to distinguishing various diseases that caused gallbladder wall thickening in the Asian population, which has good clinical value and can improve the detection rate of malignant tumors in patients with gallbladder wall thickening.

## 1 Introduction

Gallbladder cancer is the most commonly observed tumor of the biliary system and is associated with poor prognosis and a low 5-year survival rate (1). In the early stages, gallbladder cancer does not present with any specific symptoms. By the time gallbladder cancer is diagnosed, the disease has often already progressed to an advanced stage, making it difficult to achieve the desired treatment outcomes. If gallbladder cancer is diagnosed correctly in the early stages, the 5-year survival rate will significantly improve (2, 3). Research reports indicate that the occurrence of gallbladder cancer demonstrates certain geographical variations, with high incidence rates observed in Asian populations (4). The most common imaging manifestation of gallbladder cancer is gallbladder wall thickening; however, benign gallbladder diseases such as adenomyosis and polyps can also cause gallbladder wall thickening (5, 6). Both benign and malignant gallbladder diseases may present with symptoms such as upper abdominal pain and jaundice, and distinguishing benign from malignant gallbladder diseases based solely on clinical symptoms is difficult (7). Accurate diagnosis of benign and malignant gallbladder wall thickening is particularly important for the appropriate treatment of patients, avoiding overtreatment of benign diseases and delays in the treatment of malignant tumors (8, 9).

Two-dimensional transabdominal ultrasonography is the most commonly employed imaging modality for gallbladder examination. Ultrasonography is characterized by real-time dynamics, accuracy (AC), economy, and the absence of radiological damage. Compared to computed tomography (CT), transabdominal ultrasound has the advantage of avoiding exposure to nuclear radiation. Compared to transabdominal ultrasound, magnetic resonance imaging studies can also be dynamic; however, their availability is limited and they are time-consuming (10). Although two-dimensional transabdominal ultrasound is considered the preferred screening method for gallbladder wall thickening, a certain degree of inter-examiner subjectivity and a lack of consensus exists, both domestically and internationally. With the continuous development of ultrasound technology, color Doppler flow imaging (CDFI) can display the blood flow status of lesions, improving the AC of gallbladder lesion diagnosis (6). Ultrasonographic features of gallbladder wall thickening in benign diseases usually exhibit diffuse symmetrical wall thickening, whereas ultrasonographic features of gallbladder wall thickening in malignant diseases usually involve focal asymmetrical thickening without a layered appearance, destruction of the gallbladder wall, or an indistinct interface with the adjacent liver parenchyma (9, 11). In February 2022, the American journal “Abdominal Radiology” published the Gallbladder Reporting and Data System (GB-RADS) for risk stratification of gallbladder wall thickening detected on ultrasonography. The system aims to assess the risk of malignancy in gallbladder wall thickening by applying consistent ultrasound terminology, which would allow for standardized and consistent descriptions of the lesion characteristics for consistent follow-up and management in clinical practice (12).

The purpose of this study was to explore the diagnostic value of GB-RADS in distinguishing benign from malignant gallbladder wall thickening in Asian populations and to evaluate whether incorporating CDFI to GB-RADS would improve the diagnostic performance of the system, thus providing valuable diagnostic methods for patients with gallbladder wall thickening.

## 2 Methods

### 2.1 Materials

**Figure 1:**
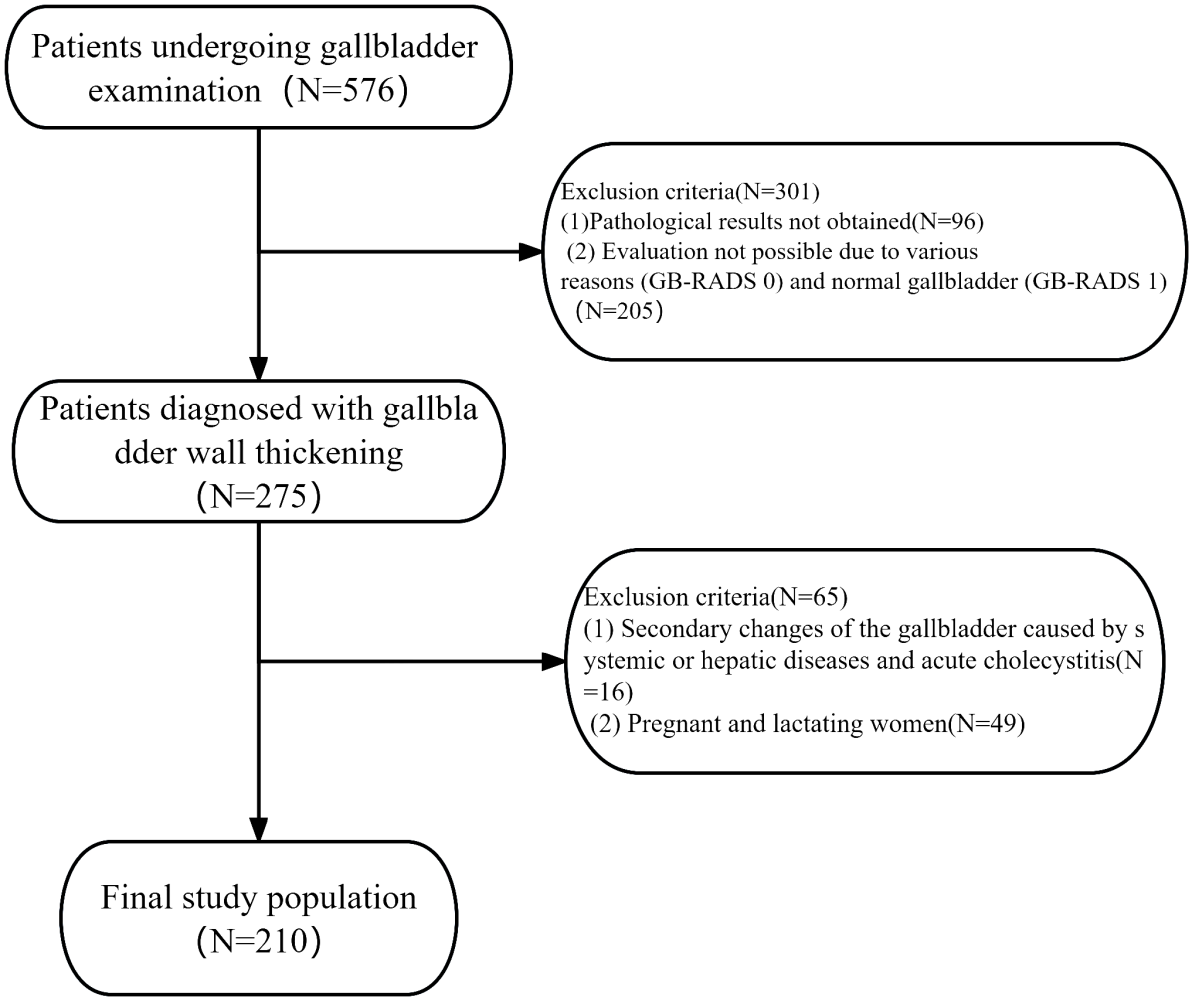
Flowchart showing a selection of patients with gallbladder wall thickening.

This retrospective study was approved by the hospital’s Institutional Health Research Ethics Committee, and the need for informed consent was waived (ethics number: KYLL-KS-2023148). Ultrasound images of all patients with gallbladder wall thickening from the hospital’s local picture archiving and communication system between February 2019 and May 2023 were retrospectively analyzed. The patient selection flowchart is displayed in **Figure 1**. The inclusion criteria were as follows: (1) availability of preoperative gallbladder ultrasonography and postoperative pathological results; (2) thickening of the gallbladder wall observed using abdominal ultrasound (gallbladder wall thickness > 3 mm); (3) no radiotherapy, chemotherapy, immunotherapy, or other related treatments before the examination; and (4) age > 18 years. The exclusion criteria were as follows: (1) pathological results not obtained; (2) evaluation impossible for various reasons (GB-RADS 0) and normal gallbladder (GB-RADS 1); (3) secondary changes in the gallbladder caused by systemic or hepatic diseases and acute cholecystitis; and (4) pregnant and lactating women.

### 2.2 Methods

Diagnostic color ultrasound machines, such as PHILIPS IU22, TOSHIBA Aplio 500, and Canon Apilo i800, were used in this study. Gallbladder ultrasonography was performed after 6 hours of fasting. Ultrasonography of the left lateral position of the gallbladder was performed in both the sagittal and axial planes. The imaging provided a complete assessment of the gallbladder from multiple perspectives. The examination primarily focused on the following morphological characteristics for each evaluated gallbladder: the thickness of the gallbladder wall, extent, and symmetry of the gallbladder wall thickening, site of the gallbladder wall thickening, presence of contents in the gallbladder cavity, presence of a layering phenomenon in the gallbladder wall, intramural changes in the wall, clear interface with the liver, and distribution of CDFI in the gallbladder wall thickening. Two experts with over 10 years of experience in abdominal ultrasound diagnosis were trained in the GB-RADS. They classified each lesion under the assumption of unknown pathology, and when disagreements occurred, they were resolved through re-examination and discussion.

The GB-RADS grading system was divided into six classes ranging from 0 to 5, including a range from assessable to high-risk. GB-RADS 0 signifies cases that cannot be evaluated due to technical reasons and patient-related factors such as obesity, porcelain gallbladder, and the presence of gas in the gallbladder lumen. GB-RADS 1 indicates a normal gallbladder, adequate gallbladder distension, and the gallbladder wall thickness ≤3 mm; GB-RADS 2 encompasses benign conditions (probability of malignancy < 2%), including (1) symmetric circumferential thickening with or without intramural changes or focal thickening with intramural changes, (2) layered appearance, (3) clear interface with the liver; GB-RADS 3 suggests a suspected malignant tumor (probability of malignancy 2%–50%), including (1) circumferential thickening without layered appearance, (2) no intramural features (localized thickening of cysts or echo lesions), (3) clear interface with the liver; GB-RADS 4 includes a lesion suggestive of malignant tumor (probability of malignant 50%–90%), presenting circumferential or focal thickening without stratification and with a vague interface with the life; GB-RADS 5: indicates a high suspicion of malignancy (probability of malignancy >90%), this included the features of GB-RADS 4, with the addition of definite extrahepatic invasion of the gallbladder wall. Moreover, invasion characteristics may involve the direct extension of the biliary or vascular structures by the mural thickening, as well as adjacent liver mass with the mural thickening (12). Color Doppler blood flow grading was divided into grades 0 to III according to Adler’s blood flow grading (13): 0, no flow signal; I, a small flow signal with one or two dots and short rods; II, moderate flow with three or four dots or one main blood vessel; and III, abundant flow with more than four dots or two main blood vessels. When the CDFI of the lesion in GB-RADS 3 was grade 0–I, the original grading remained unchanged and was represented as GB-RADS+CDFI 3. When the CDFI was grade II–III, it was adjusted upward to one grade, as demonstrated by GB-RADS+CDFI 4. When the CDFI of the lesion in GB-RADS 4 was grade 0–I, it was adjusted downward to one grade, as demonstrated by GB-RADS+CDFI 3. Furthermore, when the CDFI of the lesion in GB-RADS 4 was grade 0–I, a downward adjustment of one grade was represented by GB-RADS+CDFI 3. When the CDFI of the lesion in GB-RADS 4 was grades II–III, the original grading remained unchanged, as indicated by GB-RADS+CDFI 4. The lesions in GB-RADS 3 did not have a downward adjustment, as demonstrated by GB-RADS+CDFI 2, while the lesions in GB-RADS 5 did not have an upward adjustment, as indicated by GB-RADS+CDFI 5 (14, 15).

### 2.3 Statistical analysis

Data were analyzed using SPSS 26.0 and MedCalc 19.0. The age of the patients in the malignant and benign groups conformed to the normal distribution and was expressed as mean ± standard deviation, and comparisons were made using the independent samples t-test. The ultrasonographic features of the gallbladder and CDFI distributions of the gallbladder in the malignant and benign groups were expressed as numbers and percentages. Additionally, comparisons were made using the chi-square test. Sensitivity (SE), specificity (SP), positive predictive value, negative predictive value, AC, and Jordon indices were calculated for GB-RADS and GB-RADS combined with CDFI. For statistical convenience, intraepithelial neoplasia was classified as a malignant tumor in this study, and pathological findings were used as the gold standard to generate the receiver operating characteristic (ROC) curve, determine the optimal cutoff value, and calculate the area under the curve (AUC). Differences between the AUC values were analyzed using McNemar’s test. A P-value < 0.05 was considered statistically significant. Kappa values were used to assess inter-observer agreement (IRA) between the two observers for GB-RADS. Moreover, 0.21 ≤ Kappa ≤ 0.40 indicated general consistency, 0.41 ≤ Kappa ≤ 0.60 was suggestive of moderate consistency, 0.61 ≤ Kappa ≤ 0.80 signified good consistency, and 0.81 ≤ Kappa ≤ 1.00 was suggestive of excellent consistency.

## 3 Results

### 3.1 General characteristics

The study included 210 patients who underwent surgery and whose pathological results were obtained. The age range was between 29 and 84 years, with an average age of 59.66 ± 13.37. The average age in the benign group was 56.14 ± 13.43, while that in the malignant groups was 65.38 ± 11.18. According to the pathological results, the study included 130 cases of benign disease and 80 cases of malignant disease, with cholecystitis and adenocarcinoma being the most common benign and malignant diseases, respectively. The analysis encompassed 44 cases of GB-RADS 2, 76 cases of GB-RADS 3, 76 cases of GB-RADS 4, and 14 cases of GB-RADS 5. According to GB-RADS and CDFI, 44 cases of GB-RADS+CDFI 2, 85 cases of GB-RADS+CDFI 3, 67 cases of GB-RADS+CDFI 4, and 14 cases of GB-RADS+CDFI 5 were present. The specific pathological results and classifications are presented in **Table 1**.

**Table 1:** GB-RADS and pathology.

Pathology	GB-RADS				N
	2	3	4	5	
Chronic cholecystitis	30	66	8	0	104
Adenomyosis of the gallbladder	10	4	0	0	14
Xanthogranulomatous cholecystitis	0	0	12	0	12
Intraepithelial neoplasia	4	6	6	0	16
Adenocarcinoma	0	4	38	4	46
Adenosquamous carcinoma	0	0	2	0	2
Papillary carcinoma	0	0	6	0	6
Metastatic tumor	0	0	0	10	10
N	44	80	72	14	210

The meaning of N is the sample size of the patients.

### 3.2 Univariate and multivariate analysis of ultrasound characteristics

**Table 2** displays the univariate and multivariate analyses of the ultrasound characteristics related to gallbladder wall thickening. In the univariate analysis, significant differences (P < 0.05) were observed between the benign and malignant groups in the extent of gallbladder wall thickening, symmetry of gallbladder wall thickening, the presence of layered appearance in the gallbladder wall, intramural changes in the gallbladder wall, whether the interface with the liver was clear, and the distribution of CDFI in the gallbladder wall thickening. Multivariate analysis of the aforementioned characteristics revealed that an unclear interface with the liver and grade III–IV of CDFI were associated with the development of malignant tumors of the gallbladder (all P < 0.05).

**Table 2:** Ultrasonographic features of gallbladder wall thickening.

Ultrasonographic features	Pathology			Univariate analysis		multivariate analysis	
	Benign (N=130)		Malignant(N=80)	χ^2^	P value	P value	OR (95%CI)
Symmetry of wall thickening	yes	108	38	29.584	<0.001	0.082	0.231(0.044,1.207)
	no	22	42				
Extent of wall thickening	diffuse	94	44	6.584	0.010	0.686	0.713(0.138,3.678)
	part	36	36				
Layered appearance	Yes	40	4	19.856	<0.001	0.102	7.057(0.679,73.353)
	no	90	76				
Intramural changes (foci of intramural echogenicity and cysts)	existent	34	6	11.176	0.001	0.149	0.220(0.028,1.716)
	non-existent	96	74				
Interface with liver	clear	128	66	17.926	<0.001	0.001	0.016(0.001,0.365)
	unclear	2	14				
Blood grading	I-II	127	7	169.649	<0.001	<0.001	0.836(0.530,6.235)
	III-IV	3	73				

### 3.3 Diagnostic performance of GB-RADS, and GB-RADS combined with CDFI

**Figure 2:**
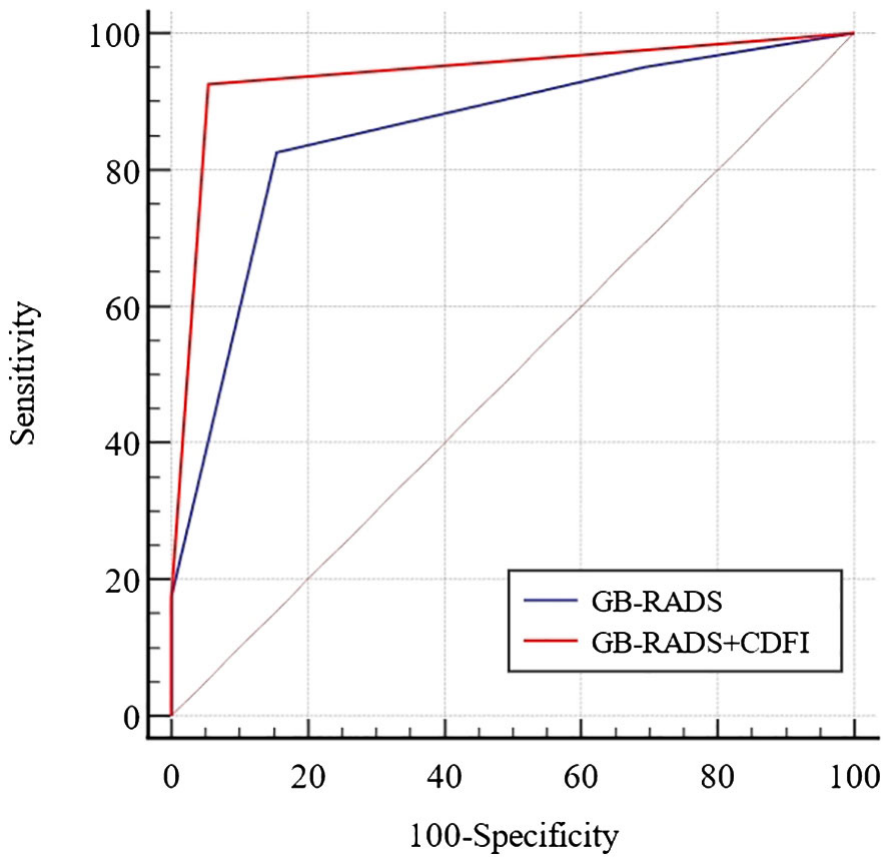
Receiver-operator characteristic curves for diagnosis of benign and malignant gallbladder wall thickening by GB-RADS and GB-RADS combined with CDFI.

**Table 3** demonstrates the incidence of malignancy in GB-RADS, and the risks of malignancy in GB-RADS 2, 3, 4, and 5 were 9%, 12.5%, 72.2%, and 100%, respectively, with statistically significant differences (P < 0.001). The ROC curves of the GB-RADS and GB-RADS combined with CDFI for the diagnosis of gallbladder malignancy are illustrated in **Figure 2**. The Jordon index of GB-RADS was 0.671; the AUC was 0.855 (95% CI: 0.800–0.900). The best cut-off value for predicting gallbladder malignancy was determined to be greater than GB-RADS 3. The Jordon index of GB-RADS combined with CDFI was 0.908 with an AUC of 0.965 (95% CI: 0.930–0.985), and the AUC of GB-RADS combined with CDFI for diagnosing gallbladder malignancy was higher than that of GB-RADS alone, with a statistically significant difference (P=0.0001). The diagnostic performances of GB-RADS and GB-RADS combined with CDFI for malignant gallbladder tumors are demonstrated in **Table 4**. The study identified that the SE, SP, and AC of the GB-RADS alone in diagnosing malignant gallbladder tumors were 82.5%, 84.6%, and 83.8%, respectively. However, when GB-RADS was combined with the CDFI, the SE, SP, and AC improved to 96.2%, 94.6%, and 95.2%, respectively. This indicated that the combination of the GB-RADS and CDFI was more effective in diagnosing malignant gallbladder tumors than the GB-RADS alone. Importantly, these differences in diagnostic AC were statistically significant (all P < 0.05).

**Table 3:** Incidence of malignancy in GB-RADS.

Category	Total	Final Diagnosis		Calculated malignancy rate (%)	Recommended malignancy rate (%)	P value
		Benign (n = 469)	Malignant (n = 80)			
GB-RADS 2	44	40	4	9%	<2%	<0.001
GB-RADS 3	80	70	10	12.5%	2%~50%	
GB-RADS 4	72	20	52	72.2%	50%~90%	
GB-RADS 5	14	0	14	100%	<90%	

**Table 4:** Diagnostic performance of GB-RADS and GB-RADS combined with CDFI for benign and malignant gallbladder wall thickening.

	AUC	SE	SP	PPV	NPV	AP
GB-RADS	0.855	82.5	84.6	76.7	88.7	83.8
GB-RADS+CDFI	0.965	96.2	94.6	91.7	97.6	95.2
χ^2^/Z	3.941	7.964	6.985	7.083	7.287	16.623
P	0.0001	0.005	0.008	0.008	0.005	<0.001

SE, Sensitivity; SP, Specificity; PPV, Positive predictive value; NPV, Negative predictive value; AC, Accuracy.

### 3.4 IRA between the two observers

Two observers independently analyzed the ultrasound images using the GB-RADS, and their detailed categorization results are presented in **Table 5**. The Kappa value was 0.870 (95% CI: 0.823–0.917), with P < 0.001, indicating an excellent level of agreement between the observers.

**Table 5:** GB-RADS of inter-observer agreement.

		Observer 2				
		GB-RADS 2	GB-RADS 3	GB-RADS 4	GB-RADS 5	Total
Observer 1	GB-RADS 2	50	10	0	0	60
	GB-RADS 3	10	54	3	0	67
	GB-RADS 4	0	4	67	0	71
	GB-RADS 5	0	0	0	12	12
	Total	60	68	70	12	210

**Figure 3:**
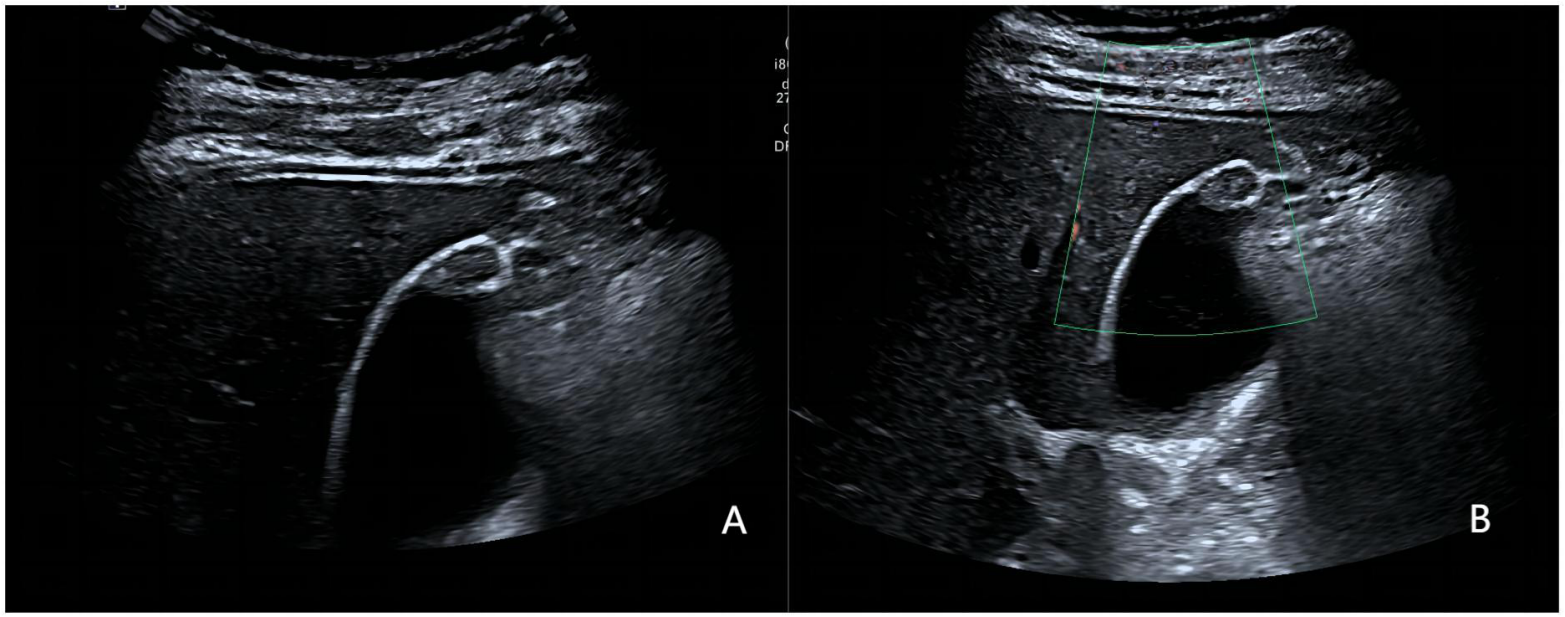
A 36-year-old woman with pathologically proven xanthogranulomatous cholecystitis. **(A)** Focak thickening of the base of the gallbladder with layering; **(B)** CDFI=0.Based on a consensus review of the sonographic findings, the lesion was categorized as GB-RADS 2, GB-RADS+CDFI 2.

**Figure 4:**
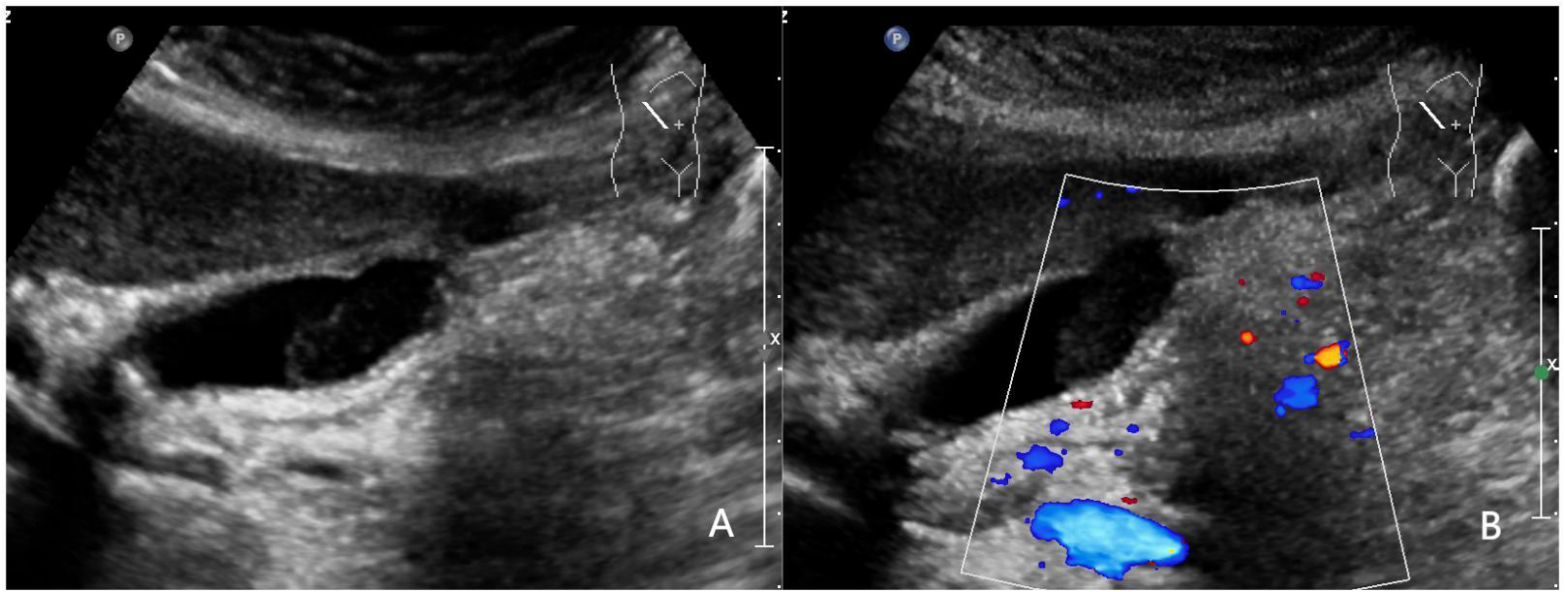
A 52-year-old woman with pathologically proven chronic cholecystitis. **(A)** Focal thickening of the base of the gallbladder, with no stratified appearance, and loss of interface with the liver.; **(B)** CDFI=0.Based on a consensus review of the sonographic findings, the lesion was categorized as GB-RADS 4, GB-RADS+CDFI 3.

**Figure 5:**
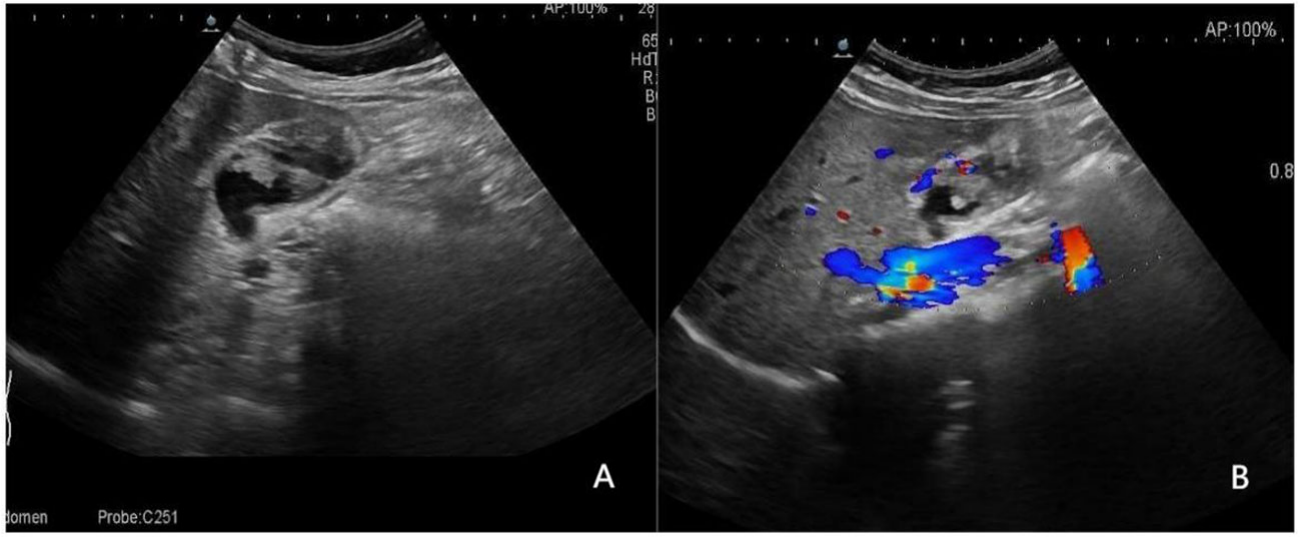
A 52-year-old man with a pathologically proven adenocarcinoma. **(A)** Focal thickening of the base of the gallbladder, with no stratified appearance, and loss of interface with the liver.; **(B)** CDFI=II. Based on a consensus review of the sonographic findings, the lesion was categorized as GB-RADS 4, GB-RADS+CDFI 4.

Representative cases from our study are displayed in **Figures 3**–**5**.

## 4 Discussion

Thickening of the gallbladder wall is a common manifestation of biliary system diseases. In the presence of a history of right upper abdominal pain and inflammatory findings, thickening of the gallbladder wall usually is associated with acute cholecystitis. However, in the absence of clinical symptoms of cholecystitis, consideration should be given to various perspectives, especially gallbladder cancer (16). Gallbladder cancer presents with no obvious symptoms during the early stages, progresses rapidly, and is prone to recurrence and metastasis (17). These characteristics have a significant impact on a patient’s treatment and lead to an extremely poor overall prognosis. Therefore, an early and accurate diagnosis of gallbladder wall thickening is extremely important for the effective management and favorable prognosis of patients. Conventional ultrasound is currently considered the preferred imaging modality for the clinical diagnosis of gallbladder diseases (18). However, no consensus exists on how to differentiate between benign and malignant gallbladder wall thickening. The GB-RADS is a risk stratification system that categorizes gallbladder wall thickening based on ultrasound characteristics and adopts different management measures for each category, helping to improve diagnostic AC (12). To date, the performance of GB-RADS has not been validated. This study analyzed the diagnostic value of GB-RADS in diagnosing gallbladder wall thickening in an Asian population and evaluated whether the addition of CDFI can improve the diagnostic performance of GB-RADS.

This study discovered that the ROC of GB-RADS for the diagnosis of malignant gallbladder cancer was 0.855. When using a GB-RADS score > 3 as a predictive factor for malignant tumors, the SE was 82.5% and the SP was 84.6%, indicating good diagnostic performance. In routine ultrasound diagnosis of malignant gallbladder cancer, the SE was 65.3%–82.5%, with significant variation. Some researchers have explored the value of high-frame-rate contrast-enhanced ultrasound for gallbladder wall thickening in nonacute settings (19). The study determined that the SE, SP, and AC of the GB-RADS for diagnosing malignant gallbladder cancer were only 68.75%, 73.33%, and 71.74%, respectively, which were lower than those in our study. The reason for this difference may be that, in the study by Zhu et al., the sample size consisted of 46 cases, whereas in our study, the sample size comprised 210. Despite being derived from routine transabdominal ultrasonography, the GB-RADS identifies key ultrasonographic features for each risk category (20–23). The ultrasonic features adopt standard terminology and describe the meanings of these terms. Management strategies for malignant potential were proposed for each risk category. This may explain why GB-RADS exhibits high SE and SP. When the GB-RADS was combined with the CDFI for the diagnosis of gallbladder cancer, the AUC, SE, and SP were 0.965, 96.2%, and 94.6%, respectively. The AUC, SE, and SP for differentiating benign and malignant gallbladder wall thickening using the GB-RADS combined with CDFI were all higher than those obtained when the GB-RADS was used alone. Additionally, CDFI can distinguish between benign and malignant disease by assessing blood supply status at the lesion site (6, 24). Studies have indicated that most malignant lesions exhibit blood flow signals, whereas in benign lesions they are hardly observed (25). The pathological basis of gallbladder cancer with abundant blood flow is the abnormal proliferation of blood vessels within the tumor, thickening, and dilation of the gallbladder artery and its branches, and a tortuous and disorderly blood vessel shape, which in turn increases blood flow (25, 26). Some researchers have reported elevated SE and SP in detecting blood flow signals, particularly in distinguishing early malignant lesions of the gallbladder from benign gallbladder bulge lesions. This underscores the effectiveness of CDFI as a method for determining the benign or malignant nature of gallbladder bulge lesions (27). Combining CDFI with the GB-RADS enhances the diagnostic AC in differentiating between benign and malignant gallbladder wall thickening disease in Asian populations. This approach evaluates ultrasound characteristics using two-dimensional ultrasound alongside CDFI, significantly improving diagnostic performance (28).

We analyzed the ultrasonographic features associated with gallbladder wall thickening. The results demonstrated that an unclear interface with the liver, grades III–IV of CDFI, was associated with gallbladder malignancy (P < 0.05), confirming the role of key ultrasound terms in the GB-RADS. Our findings suggest that only three of the 76 cases with pathologic grades III–IV were benign, and grades III–IV of the CDFI were associated with gallbladder malignancy in both univariate and multivariate analyses. Therefore, the addition of CDFI to GB-RADS may improve the diagnostic performance of the system. In the current study, we evaluated the incidence of malignant tumors across different GB-RADS categories and discovered that the incidence of malignant tumors in GB-RADS 3, 4, and 5 aligned with the recommended rate, whereas the incidence of malignant tumors in G-RADS 2 exceeded the recommended rate. A possible explanation for this observation could be that in the cases included in this study, the ultrasound findings of intraepithelial neoplasia did not display any malignant features. Therefore, based on the GB-RADS, they were classified as GB-RADS 2. However, in the present study, intraepithelial neoplasia was classified as a malignant tumor. Therefore, further research is required to assess more reasonable risks. False-positive cases mainly occurred in GB-RADS 4, including eight cases of chronic cholecystitis and 12 cases of xanthogranulomatous cholecystitis. All ultrasounds demonstrated diffuse thickening of the gallbladder wall with loss of normal structure, inhomogeneous internal echoes, and an unclear interface with the liver, These findings were indicative of a gallbladder tumor invading the liver, leading to classification as GB-RADS 4. When CDFI was added for diagnosis, the blood flow signals within most lesions were insignificant and could be downgraded.

The consistency and reproducibility of GB-RADS are also very important. During our analysis, we evaluated the IRA between the two experts using the kappa statistic. The obtained kappa value of 0.870 revealed a high level of agreement and excellent consistency in the application of the GB-RADS. These results demonstrate that the GB-RADS can be reliably and consistently implemented by experienced professionals in the field of diagnostic ultrasound.

Our study has some limitations. First, this study was retrospective in nature; we analyzed static images retained in the system, which may resulted in some errors in diagnostic AC. Furthermore, ultrasound images are manipulated by different operators using different ultrasound machinery with varying image qualities, which may have caused selection bias. Therefore, future multicenter, large-sample prospective studies are needed to validate the diagnostic performance of GB-RADS. Furthermore, several studies have demonstrated that the implementation of Artificial Intelligence (AI) has the potential to enhance the AC and precision of ultrasound in distinguishing between benign and malignant tumors. Combining AI with the GB-RADS holds great promise for improving the overall diagnostic performance in detecting and diagnosing gallbladder cancer (29).

In conclusion, GB-RADS combined with CDFI has high SE and SP to distinguish between benign and malignant gallbladder wall thickening in Asian populations, indicating the potential of the system to significantly enhance the AC of diagnosing gallbladder diseases. Moreover, the GB-RADS displayed good consistency in its findings, further validating its clinical utility. To ensure the widespread applicability and reliability of this approach, future research should focus on conducting multicenter, large-sample prospective studies that would assist healthcare professionals in effectively assessing and managing gallbladder diseases, leading to improved patient outcomes and enhanced medical decision-making.

## Data availability statement

The raw data supporting the conclusions of this article will be made available by the authors, without undue reservation.

## Ethics statement

The studies involving humans were approved by Medical Ethics Committee of Qilu Hospital of Shandong University (Qingdao). The studies were conducted in accordance with the local legislation and institutional requirements. The ethics committee/institutional review board waived the requirement of written informed consent for participation from the participants or the participants’ legal guardians/next of kin because The study involved only ultrasound images of patients. Written informed consent was not obtained from the individual(s) for the publication of any potentially identifiable images or data included in this article because The study involved only ultrasound images of patients.

## Author contributions

RW: Conceptualization, Data curation, Formal analysis, Methodology, Validation, Writing – original draft, Writing – review & editing. LLv: Writing – review & editing. LLi: Writing – review & editing.

## References

[1] Lazcano-Ponce EC Miquel JF Muñoz N Herrero R Ferrecio C Wistuba II. Epidemiology and molecular pathology of gallbladder cancer. CA Cancer J Clin. (2001) 51:349–64. doi: 10.3322/canjclin.51.6.349 11760569

[2] Sharma A Sharma KL Gupta A Yadav A Kumar A. Gallbladder cancer epidemiology, pathogenesis and molecular genetics: recent update. World J Gastroenterol. (2017) 23:3978–98. doi: 10.3748/wjg.v23.i22.3978 PMC5473118 28652652

[3] Gupta P Kumar M Sharma V Dutta U Sandhu MS. Evaluation of gallbladder wall thickening: A multimodality imaging approach. Expert Rev Gastroenterol Hepatol. (2020) 14:463–73. doi: 10.1080/17474124.2020.1760840 32323586

[4] Misra S Chaturvedi A Misra NC Sharma ID. Carcinoma of the gallbladder. Lancet Oncol. (2003) 4:167–76. doi: 10.1016/s1470-2045(03)01021-0 12623362

[5] Yu MH Kim YJ Park HS Jung SI. Benign gallbladder diseases: imaging techniques and tips for differentiating with Malignant gallbladder diseases. World J Gastroenterol. (2020) 26:2967–86. doi: 10.3748/wjg.v26.i22.2967 PMC7304100 32587442

[6] Zhu L Han P Jiang B Zhu Y Li N Fei X. Value of micro flow imaging in the prediction of adenomatous polyps. Ultrasound Med Biol. (2023) 49:1586–94. doi: 10.1016/j.ultrasmedbio.2023.03.004 37012096

[7] Chatterjee A Lopes Vendrami C Nikolaidis P Mittal PK Bandy AJ Menias CO. Uncommon intraluminal tumors of the gallbladder and biliary tract: spectrum of imaging appearances. Radiographics. (2019) 39:388–412. doi: 10.1148/rg.2019180164 30707646

[8] Kim SJ Lee JM Lee JY Kim SH Han JK Choi BI. Analysis of enhancement pattern of flat gallbladder wall thickening on Mdct to differentiate gallbladder cancer from cholecystitis. AJR Am J Roentgenol. (2008) 191:765–71. doi: 10.2214/ajr.07.3331 18716107

[9] Soundararajan R Marodia Y Gupta P Rana P Chhabra M Kalage D. Imaging patterns of wall thickening type of gallbladder cancer. Clin Exp Hepatol. (2022) 8:255–66. doi: 10.5114/ceh.2022.122285 PMC9850297 36683868

[10] Cheng Y Wang M Ma B Ma X. Potential role of contrast-enhanced ultrasound for the differentiation of Malignant and benign gallbladder lesions in east asia: A meta-analysis and systematic review. Med (Baltimore). (2018) 97:e11808. doi: 10.1097/md.0000000000011808 PMC6112946 30113470

[11] Rana P Gupta P Kalage D Soundararajan R Kumar MP Dutta U. Grayscale ultrasonography findings for characterization of gallbladder wall thickening in non-acute setting: A systematic review and meta-analysis. Expert Rev Gastroenterol Hepatol. (2022) 16:59–71. doi: 10.1080/17474124.2021.2011210 34826262

[12] Gupta P Dutta U Rana P Singhal M Gulati A Kalra N. Gallbladder reporting and data system (Gb-rads) for risk stratification of gallbladder wall thickening on ultrasonography: an international expert consensus. Abdom Radiol (NY). (2022) 47:554–65. doi: 10.1007/s00261-021-03360-w 34851429

[13] Adler DD Carson PL Rubin JM Quinn-Reid D. Doppler ultrasound color flow imaging in the study of breast cancer: preliminary findings. Ultrasound Med Biol. (1990) 16:553–9. doi: 10.1016/0301-5629(90)90020-D 2238263

[14] Chen L Zhan J Diao XH Liu YC Shi YX Chen Y. Additional value of superb microvascular imaging for thyroid nodule classification with the thyroid imaging reporting and data system. Ultrasound Med Biol. (2019) 45:2040–8. doi: 10.1016/j.ultrasmedbio.2019.05.001 31130409

[15] Zheng X Li F Xuan ZD Wang Y Zhang L. Combination of shear wave elastography and bi-rads in identification of solid breast masses. BMC Med Imaging. (2021) 21:183. doi: 10.1186/s12880-021-00702-4 34852775 PMC8638471

[16] Jenssen C Lorentzen T Dietrich CF Lee JY Chaubal N Choi BI. Incidental findings of gallbladder and bile ducts-management strategies: general aspects, gallbladder polyps and gallbladder wall thickening-a world federation of ultrasound in medicine and biology (Wfumb) position paper. Ultrasound Med Biol. (2022) 48:2355–78. doi: 10.1016/j.ultrasmedbio.2022.06.016 36058799

[17] Miranda-Filho A Piñeros M Ferreccio C Adsay V Soerjomataram I Bray F. Gallbladder and extrahepatic bile duct cancers in the americas: incidence and mortality patterns and trends. Int J Cancer. (2020) 147:978–89. doi: 10.1002/ijc.32863 PMC8629410 31922259

[18] Gerstenmaier JF Hoang KN Gibson RN. Contrast-enhanced ultrasound in gallbladder disease: A pictorial review. Abdom Radiol (NY). (2016) 41:1640–52. doi: 10.1007/s00261-016-0729-4 27056746

[19] Zhu L Li N Zhu Y Han P Jiang B Li M. Value of high frame rate contrast enhanced ultrasound in gallbladder wall thickening in non-acute setting. Cancer Imaging. (2024) 24:7. doi: 10.1186/s40644-023-00651-x 38191513 PMC10775603

[20] Kong WT Shen HY Qiu YD Han H Wen BJ Wu M. Application of contrast enhanced ultrasound in gallbladder lesion: is it helpful to improve the diagnostic capabilities? Med Ultrason. (2018) 20:420–6. doi: 10.11152/mu-1626 30534647

[21] Zhang HP Bai M Gu JY He YQ Qiao XH Du LF. Value of contrast-enhanced ultrasound in the differential diagnosis of gallbladder lesion. World J Gastroenterol. (2018) 24:744–51. doi: 10.3748/wjg.v24.i6.744 PMC5807677 29456413

[22] Zheng SG Xu HX Liu LN Lu MD Xie XY Wang WP. Contrast-enhanced ultrasound versus conventional ultrasound in the diagnosis of polypoid lesion of gallbladder: A multi-center study of dynamic microvascularization. Clin Hemorheol Microcirc. (2013) 55:359–74. doi: 10.3233/ch-121651 23283444

[23] Wang W Fei Y Wang F. Meta-analysis of contrast-enhanced ultrasonography for the detection of gallbladder carcinoma. Med Ultrason. (2016) 18:281–28. doi: 10.11152/mu.2013.2066.183.wei 27622402

[24] Sun Y Yang Z Lan X Tan H. Neoplastic polyps in gallbladder: A retrospective study to determine risk factors and treatment strategy for gallbladder polyps. Hepatobiliary Surg Nutr. (2019) 8:219–27. doi: 10.21037/hbsn.2018.12.15 PMC6561872 31245402

[25] Boddapati SB Lal A Gupta P Kalra N Yadav TD Gupta V. Contrast enhanced ultrasound versus multiphasic contrast enhanced computed tomography in evaluation of gallbladder lesions. Abdom Radiol (NY). (2022) 47:566–75. doi: 10.1007/s00261-021-03364-6 34874479

[26] Zhou W Li G Ren L. Triphasic dynamic contrast-enhanced computed tomography in the differentiation of benign and Malignant gallbladder polypoid lesions. J Am Coll Surg. (2017) 225:243–8. doi: 10.1016/j.jamcollsurg.2017.04.014 28455251

[27] Kim SY Cho JH Kim EJ Chung DH Kim KK Park YH. The efficacy of real-time colour doppler flow imaging on endoscopic ultrasonography for differential diagnosis between neoplastic and non-neoplastic gallbladder polyps. Eur Radiol. (2018) 28:1994–2002. doi: 10.1007/s00330-017-5175-3 29218621

[28] Yang L Lin N Wang M Chen G. Diagnostic efficiency of existing guidelines and the ai-sonic™ Artificial intelligence for ultrasound-based risk assessment of thyroid nodules. Front Endocrinol (Lausanne). (2023) 14:1116550. doi: 10.3389/fendo.2023.1116550 36875473 PMC9975494

[29] Gupta P Basu S Rana P Dutta U Soundararajan R Kalage D. Deep-learning enabled ultrasound based detection of gallbladder cancer in northern India: A prospective diagnostic study. Lancet Regional Health - Southeast Asia. (2023). doi: 10.1016/j.lansea.2023.100279 PMC11096661 38756152

